# High quality genome assembly and annotation (v1) of the eukaryotic freshwater microalga *Coccomyxa elongata* SAG 216-3b

**DOI:** 10.1093/g3journal/jkae294

**Published:** 2024-12-13

**Authors:** Anton Kraege, Edgar Chavarro-Carrero, Eva Schnell, Stefanie Heilmann-Heimbach, Kerstin Becker, Karl Köhrer, Bruno Huettel, Nafiseh Sargheini, Philipp Schiffer, Ann-Marie Waldvogel, Bart P H J Thomma, Hanna Rovenich

**Affiliations:** Institute of Plant Sciences, Department of Biology, University of Cologne, Zülpicher Straße 47b, Cologne 50674, Germany; Institute of Plant Sciences, Department of Biology, University of Cologne, Zülpicher Straße 47b, Cologne 50674, Germany; Institute of Plant Sciences, Department of Biology, University of Cologne, Zülpicher Straße 47b, Cologne 50674, Germany; Institute of Human Genetics, University Hospital of Bonn, University of Bonn, Venusberg, Sigmund-Freund, Straße 25, Bonn 53127, Germany; NGS Core Facility, Medical Faculty of the University of Bonn, University of Bonn, Venusberg-Campus 1, Bonn 53127, Germany; Cologne Center for Genomics (CCG), Medical Faculty, University of Cologne, Weyertal 115b, Cologne 50931, Germany; Biological and Medical Research Centre (BMFZ), Genomics and Transcriptomics Laboratory, Heinrich-Heine-University Düsseldorf, Universitätsstraße 1, Düsseldorf 40225, Germany; Biological and Medical Research Centre (BMFZ), Genomics and Transcriptomics Laboratory, Heinrich-Heine-University Düsseldorf, Universitätsstraße 1, Düsseldorf 40225, Germany; Max Planck Genome Centre, Max Planck Institute for Plant Breeding Research, Carl-von-Linne-Weg 10, Cologne 50829, Germany; Max Planck Genome Centre, Max Planck Institute for Plant Breeding Research, Carl-von-Linne-Weg 10, Cologne 50829, Germany; Institute of Zoology, Department of Biology, University of Cologne, Zülpicher Straße 47b, Cologne 50674, Germany; Institute of Zoology, Department of Biology, University of Cologne, Zülpicher Straße 47b, Cologne 50674, Germany; Institute of Plant Sciences, Department of Biology, University of Cologne, Zülpicher Straße 47b, Cologne 50674, Germany; Department of Biology, Cluster of Excellence on Plant Sciences (CEPLAS), Zülpicher Straße 47b, Cologne 50674, Germany; Institute of Plant Sciences, Department of Biology, University of Cologne, Zülpicher Straße 47b, Cologne 50674, Germany

**Keywords:** *Coccomyxa*, unicellular algae, long-read sequencing, genome assembly, genome annotation, Trebouxiophyceae

## Abstract

Unicellular green algae of the genus *Coccomyxa* are recognized for their worldwide distribution and ecological versatility. *Coccomyxa elongata* is a freshwater species of the *Coccomyxa simplex* clade, which also includes lichen symbionts. To facilitate future molecular and phylogenomic studies of this versatile clade of algae, we generated a high-quality genome assembly for *C. elongata* Chodat & Jaag SAG 216-3b within the framework of the Biodiversity Genomics Center Cologne (BioC^2^) initiative. A combination of long-read PacBio HiFi and Oxford Nanopore Technologies with chromatin conformation capture (Hi-C) sequencing led to the assembly of the genome into 21 scaffolds with a total length of 51.4 Mb and an N50 of 2.8 Mb. Nineteen of the scaffolds represent highly complete nuclear chromosomes delimited by telomeric repeats, while the two additional scaffolds represent the mitochondrial and plastid genomes. Transcriptome-guided gene annotation resulted in the identification of 14,811 protein-coding genes, of which 61% have annotated protein family domains and 841 are predicted to be secreted. Benchmarking universal single-copy orthologs analysis against the Chlorophyta database identified a total of 1,494 (98.4%) complete gene models, suggesting a highly complete genome annotation.

## Introduction

Green algae are photosynthesizing eukaryotic organisms that differ greatly in terms of morphology and colonize a large variety of aquatic and terrestrial habitats. Phylogenetically, green algae form a paraphyletic group that has been proposed to comprise three lineages; the Prasinodermophyta, the Chlorophyta and the Streptophyta (Li *et al*. 2020). Following the divergence of the Prasinodermophyta phylum, the Viridiplanteae lineage split into Chlorophyta and Streptophyta between 1,000 and 700 million years ago (Morris *et al*. 2018). The Streptophyta contain the land plants and the streptophyte algae, whereas the Chlorophyta encompass all other green algae grouped into 8 classes of which the Pedinophyceae, Chlorodendrophyceae, Ulvophyceae, Chlorophyceae, and Trebouxiophyceae compose the so-called “core Chlorophyta” (Leliaert *et al*. 2012; Li *et al*. 2021).

The *Coccomyxa* genus comprises coccoid unicellular green algae that belong to the class of Trebouxiophyceae. Morphologically, *Coccomyxa* spp. are characterized by irregular elliptical to globular cells that range from 6–14 × 3–6 μm in size, with a single parietal chloroplast without pyrenoids, and no flagellate stages (Schmidle 1901). Members of this genus are found in freshwater, marine, and various terrestrial habitats where they occur free-living or in symbioses with diverse hosts (Darienko *et al*. 2015; Malavasi *et al*. 2016; Gustavs *et al*. 2017). Several *Coccomyxa* species establish stable, mutualistic associations with fungi that result in the formation of complex three-dimensional architectures, known as lichens (Jaag 1933; Zoller and Lutzoni 2003; Yahr *et al*. 2015; Gustavs *et al*. 2017; Faluaburu *et al*. 2019). Others associate with vascular plants or lichens as endophytes or epiphytes, respectively (Trémouillaux-Guiller *et al*. 2002; Cao et al. 2018a, 2018b; Tagirdzhanova *et al*. 2023), and frequently occur on the bark of trees (Kulichovà *et al*. 2014; Štifterovà and Neustupa 2015) where they may interact with other microbes. One novel species was recently found in association with carnivorous plants, even though the nature of this relationship remains unclear (Sciuto *et al*. 2019). Besides, *Coccomyxa* also establishes parasitic interactions with different mollusk species affecting their filtration ability and reproduction (Gray *et al*. 1999; Vaschenko *et al*. 2013; Sokolnikova *et al*. 2016; Sokolnikova *et al*. 2022).

Unlike some of its sister species, the freshwater alga *Coccomyxa elongata* has not been observed to establish symbiotic relationships. However, it belongs to the *C. simplex* clade that contains several *Coccomyxa* species, which live as photobionts in lichen symbioses (Darienko *et al*. 2015; Malavasi *et al*. 2016; Gustavs *et al*. 2017). We have recently generated a chromosome-scale genome assembly of the lichen-associated *C. viridis* strain SAG 216-4 and found a lack of synteny with the most closely related sequenced relative *C. subellipsoidea* C-169 that was isolated on Antarctica from dried algal peat (Blanc *et al*. 2012). Whether or not this lack of synteny between previously published *Coccomyxa* genomes has biological implications will have to be addressed in future. In addition to these two genomes, a high-quality genome for a nonsymbiotic strain of *C. viridis* that was isolated from a lichen thallus is available (Tagirdzhanova *et al*. 2023). For *Coccomyxa* sp. Obi, LA000219 and SUA001 chromosome-, scaffold-, and contig-level assemblies are available on NCBI, respectively, as well as two metagenome-assembled genomes of *C. subellipsoidea*. To facilitate future molecular and phylogenomic studies of this versatile clade of algae, we here present the generation of a high-quality chromosome-scale assembly of *C. elongata* SAG 216-3b using long-read PacBio HiFi and Oxford Nanopore Technology (ONT) combined with Hi-C and its transcriptome-guided annotation.

## Materials and methods

### Sample information

*C. elongata* Chodat & Jaag SAG 216-3b was ordered from the Culture Collection of Algae at the Georg-August-University Göttingen (*Sammlung von Algenkulturen der Universität Göttingen*, international acronym SAG), Germany. The stock culture was reactivated in liquid modified Waris-H growth medium (McFadden and Melkonian 1986) with soil extract and 3× vitamins (0.15 nM vitamin B12, 4.1 nM biotin, 0.3 μM thiamine-HCl, 0.8 nM niacinamide), and maintained through regular medium replacement. Cultures were grown at ∼15 μmol photons m^−2^ s^−1^ (fluorescent light tubes: L36W/640i energy saver cool white and L58W/956 BioLux, Osram, Munich, Germany) in a 14/10 h light/dark cycle at 20°C.

### DNA and RNA extraction

Cells of a 7-week-old *C. elongata* culture were harvested over 0.8 μm cellulose nitrate filters (Sartorius, Göttingen, Germany) using a vacuum pump. Material was collected with a spatula, snap-frozen, and ground in liquid nitrogen using mortar and pestle. The ground material was used for genomic DNA extraction with the RSC Plant DNA Kit (Promega, Madison, WI, USA) using the Maxwell RSC device according to manufacturer's instructions. To prevent shearing of long DNA fragments, centrifugation was carried out at 10,000 *g* during sample preparation. Following DNA extraction, DNA fragments <10,000 bp were removed using the SRE XS kit (Circulomics, Baltimore, MD, USA) according to manufacturer's instructions. DNA quantity and quality were assessed using the Nanodrop 2000 spectrometer and Qubit 4 fluorometer with the dsDNA BR assay kit (Invitrogen, Carlsbad, CA, USA), and integrity was confirmed by gel electrophoresis. High-molecular weight DNA was stored at 4°C.

For total RNA extraction, algal cells were collected from a dense 9-day-old culture and ground in liquid nitrogen using mortar and pestle. RNA was extracted with the Maxwell RSC Plant RNA kit (Promega, Madison, WI, USA) using the Maxwell RSC device according to manufacturer's instructions. RNA quality and quantity was determined using the Nanodrop 2000 and stored at −80°C.

### Pacific biosciences high-fidelity (PacBio HiFi) sequencing

HiFi libraries were prepared with the Express 2.0 Template kit (Pacific Biosciences, Menlo Park, CA, USA) and sequenced on a Sequel II/Sequel IIe instrument with 30 h movie time. HiFi reads were generated using SMRT Link (v10; Pacific Biosciences, Menlo Park, CA, USA) with default parameters.

### ONT sequencing

Library preparation with the Rapid Sequencing Kit (SQK-626 RAD004) was performed with ∼400 ng HMW DNA according to manufacturer's instructions (Oxford Nanopore Technologies, Oxford, UK). The sample was loaded onto an R9.4.1 flow cell in a minION Mk1B device (Oxford Nanopore Technologies, Oxford, UK), which was run for 24 h. Subsequent base calling was performed using Guppy (version 630 3.1.3; Oxford Nanopore Technologies, Oxford, UK). Adapter sequences were removed using Porechop (version 0.2.4 with default settings) (Wick 2018), and the reads were self-corrected and trimmed using Canu (version 1.8) (Koren *et al*. 2017).

### Chromosome conformation capture (Hi-C) and sequencing

*C. elongata* cells from a 2-week-old culture were flash frozen in liquid nitrogen and ground using mortar and pestle. Nuclei were extracted from frozen material using CelLytic PN Isolation/Extraction Kit (Sigma Aldrich, Burlington, MA, USA) according to the manufacturer's protocol, and cross-linked with 2% formaldehyde for 20 min at room temperature. A stop solution provided in the kit was applied to quench the reaction, and then pellet was collected by centrifugation for 10 min at 2000*g*. Hi-C libraries were generated using the Arima High Coverage Hi-C kit (Arima Genomics, A410110, Carlsbad, CA, USA) according to manufacturer's instructions, and subsequently paired-end (2 × 150 bp) sequenced on a Nextseq 2000 instrument (Illumina, San Diego, CA, USA).

### RNA sequencing

Library preparation for full-length mRNASeq was performed using the NEB Ultra II Directional RNA Library Prep with NEBNext Poly(A) mRNA Magenetic Isolation Module and 500 ng total RNA as starting material, except for W-RNA Lplaty, where library prep was based on 100 ng total RNA as starting material. Sequencing was performed on an Illumina NovaSeq 6000 device with 2 × 150 bp paired-end sequencing protocol and >50 M reads per sample.

### Genome assembly

PacBio HiFi reads were assembled using Raven (v1.8.1) (Vaser and Šikić 2021) with default settings. Hi-C reads were mapped onto this assembly with Juicer (v2.0) using the “assembly” option to skip the post-processing steps and generate the merged_nodups.txt file (Durand *et al*. 2016b). For the juicer pipeline, restriction site maps were generated using the *Dpn*II (GATC) and *Hin*fI (GANTC) restriction site profile and the assembly was indexed with BWA index (v0.7.17-r1188) (Li and Durbin 2009), and used to polish the assembly using 3d-dna (v180922) (Dudchenko *et al*. 2017). Afterwards, Juicebox (v1.11.08) was used for manual genome curation according to the Hi-C pattern (Durand *et al*. 2016a). Contigs were merged to scaffolds according to the Hi-C map and Ns were introduced between contigs within scaffolds, gaps between contigs were removed and contigs were merged. Subsequently, ONT reads were mapped to the assembly using Minimap2 (v2.24-r1122) and Samtools (v1.10) and mapped reads were visualized in Integrative Genome Viewer (v2.11.2) (Robinson *et al*. 2011; Danecek *et al*. 2021; Li, 2021). Whenever gaps between contigs were spanned by at least 5 reads with a mapping quality of 30, the contigs were fused in the assembly.

Potential telomeres were identified using tapestry (v1.0.0) with “AACCCT” as telomere sequence (Davey *et al*. 2020). To check for potential contaminations, Blobtools (v1.1.1) and BLAST (v2.13.0+) were used to create a Blobplot including taxonomic annotation at genus level (Camacho *et al*. 2009; Laetsch and Blaxter 2017). To check completeness of the assembly and retrieve ploidy information, kat comp from the Kmer Analysis Toolkit (v2.4.2) was used, and results were visualized using the kat plot spectra-cn function with the -x 800 option to extend the x-axis (Mapleson et al. 2017).

### Annotation

To annotate repetitive elements in the nuclear genome, a database of simple repeats was created with RepeatModeler (v2.0.3) that was expanded with transposable elements (TEs) from the TransposonUltimate resonaTE (v1.0) pipeline (Flynn *et al*. 2020; Riehl *et al*. 2022). This pipeline uses multiple tools for TE prediction and combines the prediction output. For the prediction of repetitive elements in *C. elongata* helitronScanner, ltrHarvest, mitefind, mitetracker, RepeatModeler, RepeatMasker, sinefind, tirvish, transposonPSI, and NCBICDD1000 were used within TransposonUltimate resonaTE and TEs that were predicted by at least 2 tools were added to the database. TEclass (v2.1.3) was used for classification (Abrusán *et al*. 2009). To softmask the genome and obtain statistics on the total TE and repetitive element content in the genome, RepeatMasker (v4.1.2-p1) (Smit *et al*. 2012) was used with excln option to exclude Ns in the masking.

Gene annotation in the nuclear genome was performed making use of RNA sequencing data. To this end, the genome was indexed, and reads were mapped with HiSat2 (v2.2.1) using default settings (Kim *et al*. 2019). Afterwards, BRAKER1 (v2.1.6) was used for transcriptome-guided gene prediction based on the RNA sequencing data with default settings (Hoff *et al*. 2016). To generate protein and coding sequence files the Braker output was transformed with Gffread (v0.12.7) (Pertea and Pertea 2020). Protein family (Pfam) domain annotation was performed with InterProScan (v5.61) (Paysan-Lafosse *et al*. 2023). To estimate the number of secreted proteins, SignalP (v6.0) was run in the slow-sequential mode on the annotated proteins (Teufel *et al*. 2022). Finally, Benchmarking universal single-copy orthologs (BUSCO) (v5.3.2) was run with the Chlorophyta database (chlorophyta_odb10) to estimate the completeness of the gene annotation (Manni *et al*. 2021). The circos plot visualization of the annotation was created with R (v4.2.0) and Circilize (v0.4.14) (Gu *et al*. 2014). All software and tools used for the genome assembly and annotation are summarized in Supplementary Table 1.

Organelle genomes were annotated separately. Scaffolds were identified as organelle genomes based on their lower guanine-cytosine (GC) content and smaller size. The mitochondrial genome was annotated using MFannot (Lang *et al*. 2023) as well as GeSeq (Tillich *et al*. 2017) and the annotation was combined within the GeSeq platform. The plastid genome was annotated using GeSeq alone. The annotations were visualized using the OGDraw webserver (Greiner *et al*. 2019).

## Results and discussion

**Fig. 1.**
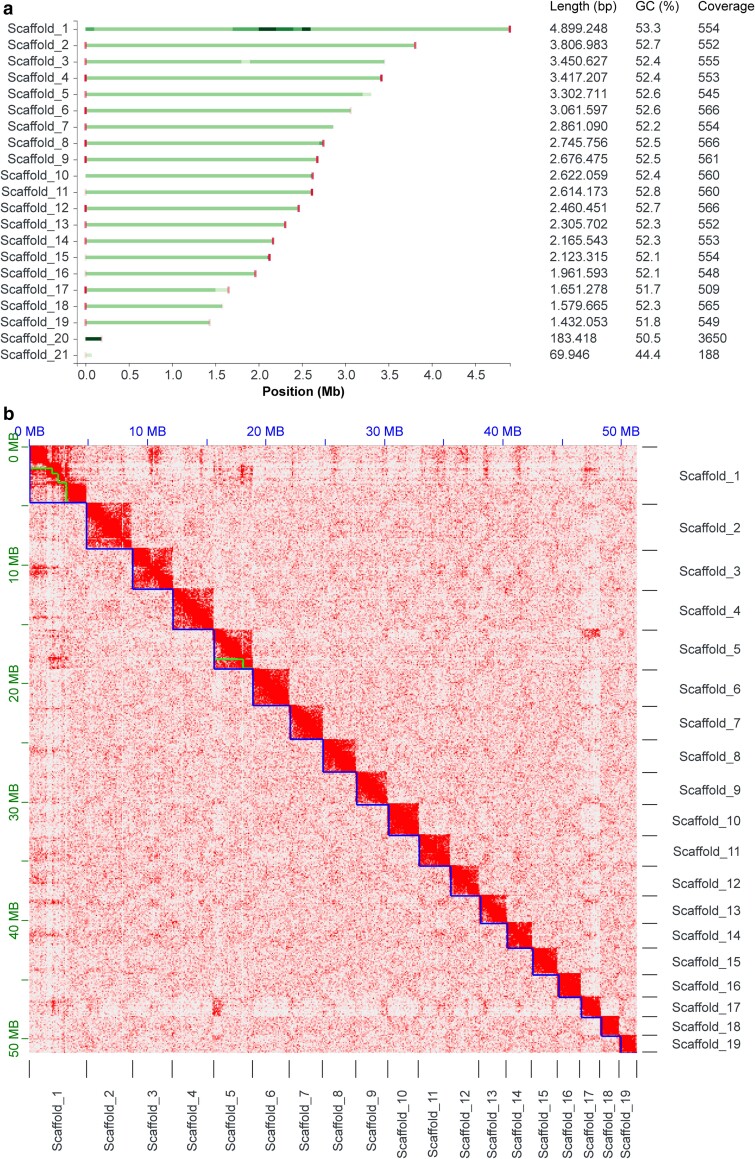
Genome assembly of *C. elongata* SAG 216-3b. a) An overview of the *C. elongata* genome assembly depicts chromosome-scale scaffolds. Green bars indicate scaffold sizes and red bars represent telomeres. Variations in color intensities correlate with read coverage. Read coverage per scaffold is determined by mapping PacBio HiFi reads onto the assembly. Scaffolds 20 and 21 represent chloroplast and mitochondrial genomes based on size and low GC contents. b) Hi-C contact map showing interaction frequencies between regions in the nuclear genome of *C. elongata*. Scaffolds are framed by blue lines while contigs within scaffolds are depicted in green.

**Fig. 2.**
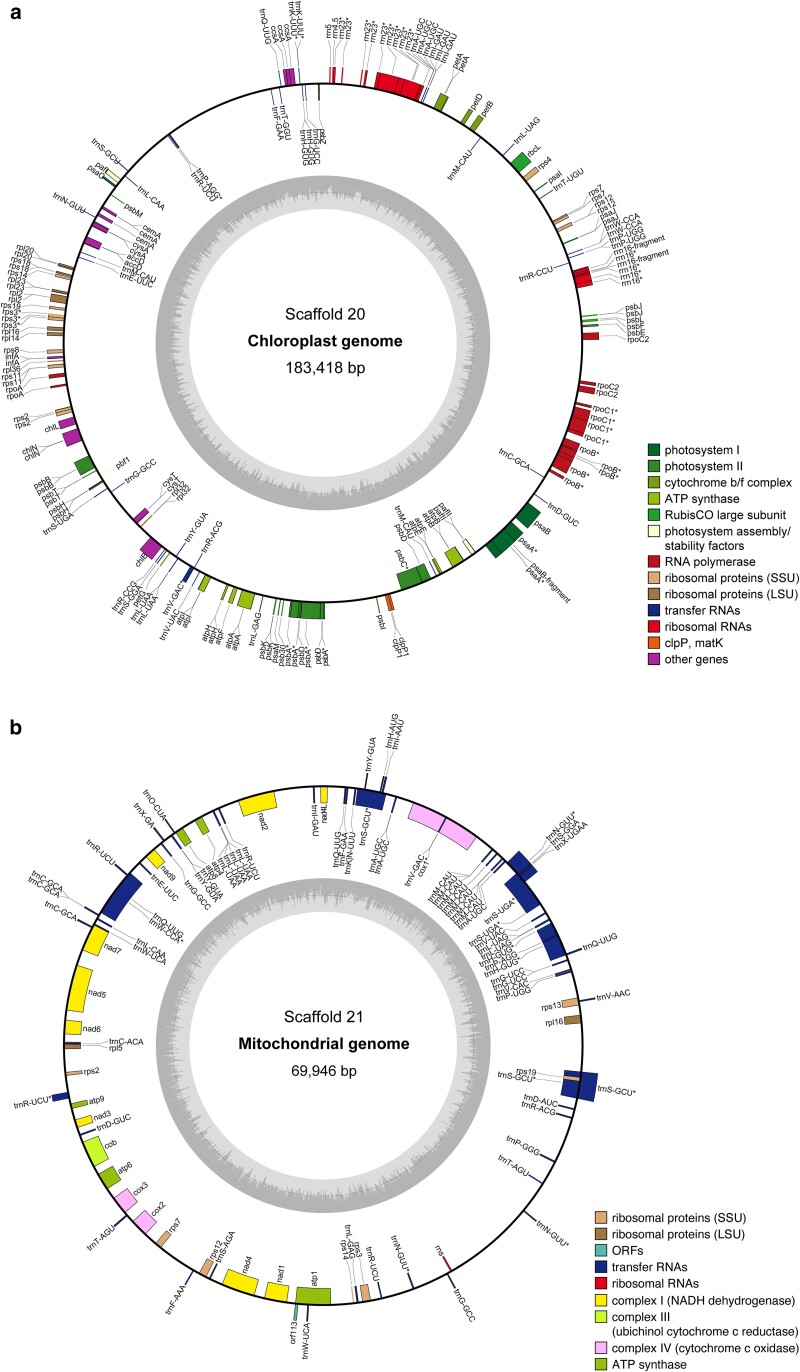
Scaffolds 20 and 21 represent the plastid and mitochondrial genomes of *C. elongata* SAG 216-3b. Gene maps of the chloroplast a) and mitochondrial b) genomes. The inner circles indicate the GC content and mapped genes are shown on the outer circles. Genes that are transcribed clockwise are placed inside the outer circles, and genes that are transcribed counterclockwise at the outside of the outer circles.

The first version of the genome of *C. elongata* was assembled from 32.2 Gbp of PacBio HiFi reads with a mean read length of 14.8 kb, 8.7 Gbp Nanopore reads with a mean read length of 8.1 kb and 2.7 million pairs of Hi-C seq data. The PacBio HiFi and ONT reads were first used to generate a hybrid assembly with Raven (Vaser and Šikić 2021), yielding 23 contigs. These contigs were scaffolded and manually curated using Hi-C data (Li and Durbin 2009; Durand *et al*. 2016a; Durand *et al*. 2016b; Dudchenko *et al*. 2017) resulting in 21 scaffolds consisting of 25 contigs with a total length of 51.4 Mb and an N50 of 2.8 Mb (Fig. 1 and Table 1). Using Tapestry (Davey *et al*. 2020), clear telomeric regions ([AACCCT]*n*) were identified at both ends of ten of the 19 genomic scaffolds (≥5 repeats) (Fig. 1a), suggesting that these represent highly complete chromosomes, which was confirmed by Hi-C analysis (Fig. 1b). Additionally, the Hi-C contact map indicated centromeres for some of the chromosomes. However, the determination of exact centromere locations on all chromosomes will require ChIP-seq analysis and CenH3 mapping. While Tapestry only identified clear telomeric sequences at one end of the nine remaining nuclear scaffolds, the Hi-C map points toward the presence of telomeric repeats at both ends of all scaffolds 1-19 (Fig. 1b). Therefore, like for *C. viridis*, this first version of the *C. elongata* genome assembly contains 19 highly complete chromosomes that compose the nuclear genome (Kraege *et al*. 2024a). Scaffolds 20 and 21 were considerably shorter than the nuclear scaffolds with ∼183 and ∼70 kb and displayed a lower GC content around 51 and 44%, respectively (Fig. 1a). This suggests that these scaffolds represent the chloroplast and mitochondrial genomes, which was confirmed by their full annotation (Fig. 2). While most green algae have smaller organelle genomes, the sizes of the *C. elongata* chloroplast and mitochondrial genomes are within the same range as those of previously described *Coccomyxa* species (Smith *et al*. 2011; Tagirdzhanova *et al*. 2023; Kraege *et al*. 2024a). Unlike *C. viridis*, however, *C. elongata* also has an unusually high GC content (>50%) in its plastid genome. For *C. subellipsoidea* high GC contents have been reported for both organelle genomes, which is thought to be due to the species' life history features (Smith *et al*. 2011).

**Table 1.** Genome features of *C. elongata* SAG 216-3b with mitochondrial and plastid genomes.

Assembly ID	*C. elongata* SAG 216-3b
Total length (bp)	51,390,890
No. of contigs	25
No. of scaffolds	21
Longest scaffold (bp)	4,899,248
N50 (bp)	2,745,756
L50	8
GC content (%)	52.46

**Fig. 3.**
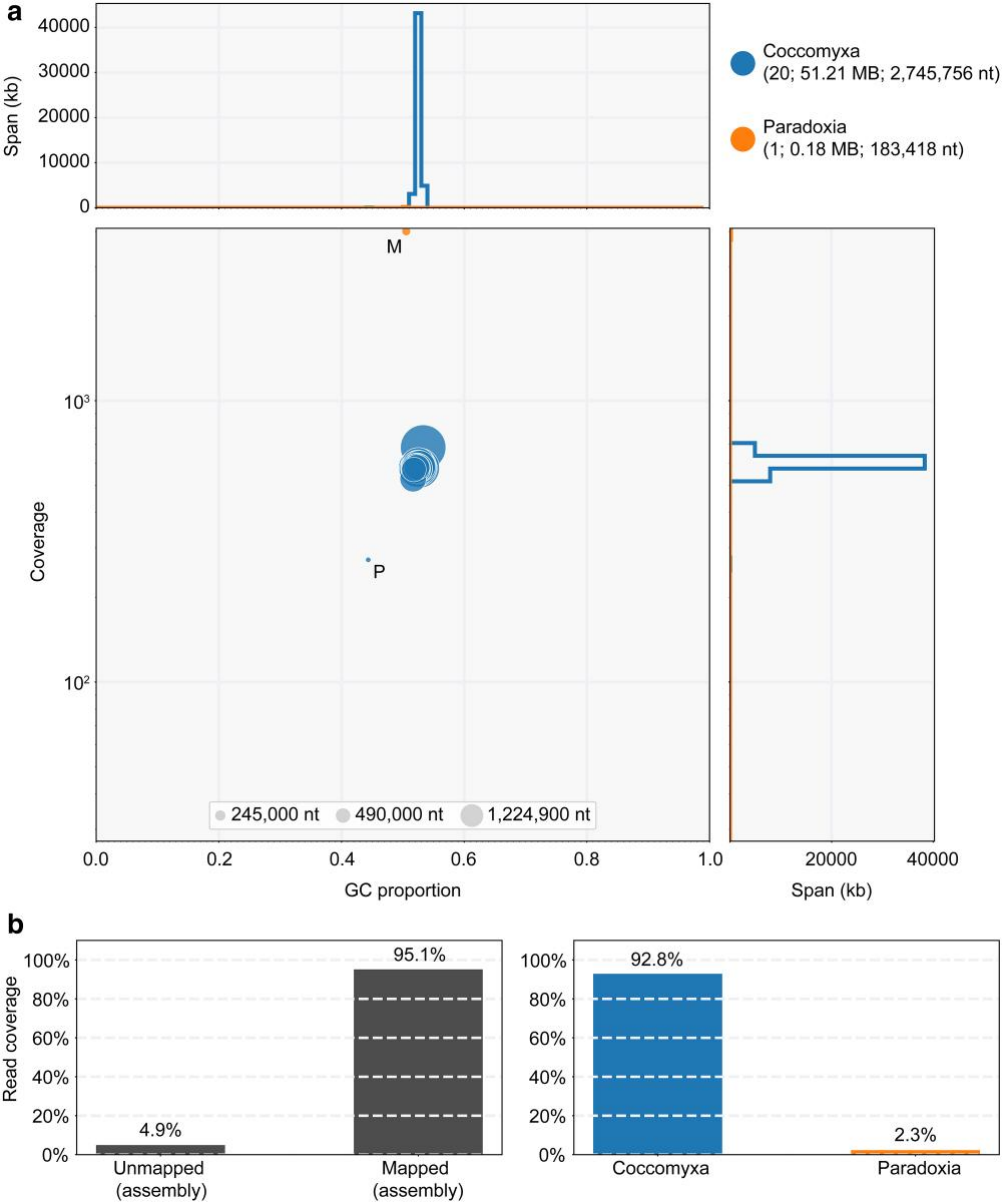
Taxonomic annotation indicates absence of contaminations in the genome assembly. a) GC coverage scatter plot (Blobplot) of the contigs from the genome assembly shows that all but one scaffolds are taxon-annotated as *Coccomyxa*. BLAST-based taxon annotation assigned the remaining scaffold, which corresponds to the mitochondrial genome (M), to the *Coccomyxa* sister genus *Paradoxia* possibly due to a short evolutionary distance between the mitochondrial genomes of their member species. All scaffolds that belong to the nuclear genome have a similar GC content (∼53%). The GC content of the mitochondrial (M) and plastid (P) genomes are lower with ∼51% and ∼44%, respectively. b) In total 95.1% of the reads can be mapped onto the assembly and 92.8% are clearly classified as *Coccomyxa*. The classification of 2.3% of the reads corresponding to the mitochondrial genome as *Paradoxia* is considered a BLAST-related artifact.

**Fig. 4.**
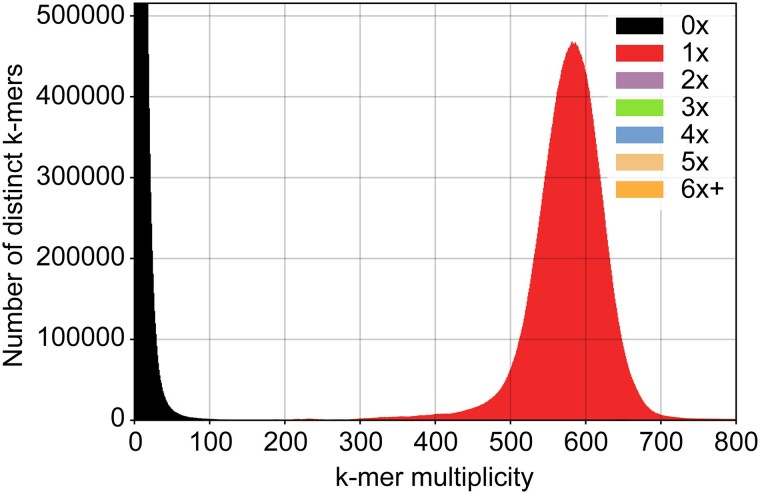
The *C. elongata* SAG 216-3b genome is haploid. The KAT spectra-cn plot depicts the 27-mer multiplicity of the PacBio HiFi reads against the genome assembly. Black areas under the peaks represent k-mers present in the reads but absent from the assembly, colored peaks indicate k-mers that are present once to multiple times in the assembly. The single peak at a k-mer multiplicity of 590 in the KAT spectra-cn plot suggests that *C. elongata* has a haploid genome, while the black peak at low multiplicity shows that the assembly is highly complete and that all reads are represented in the assembly.

To rule out the presence of contaminants, the assembly and PacBio HiFi raw reads were used to produce a Blobplot (Camacho *et al*. 2009; Laetsch and Blaxter 2017), which indicated that 92.8% of the mapped reads match only the *Coccomyxa* genus (Fig. 3). The BLAST-based taxon annotation assigned the remaining 2.3% of mapped reads to the *Coccomyxa* sister genus *Paradoxia* (Fig. 3b) (Lemieux *et al*. 2014). This could be explained by a short evolutionary distance between their mitochondrial genomes since low mitogenome copy numbers (here: 188) correlate with low substitution rates in plants (Zwonitzer *et al*. 2024). Therefore, the original sample is considered to have been free of contaminants. KAT analysis showed a single peak of k-mer multiplicity based on HiFi reads that were represented once in the assembly (Fig. 4) (Mapleson *et al*. 2017), indicating that the genome of *C. elongata* represents a high-quality, haploid genome.

**Fig. 5.**
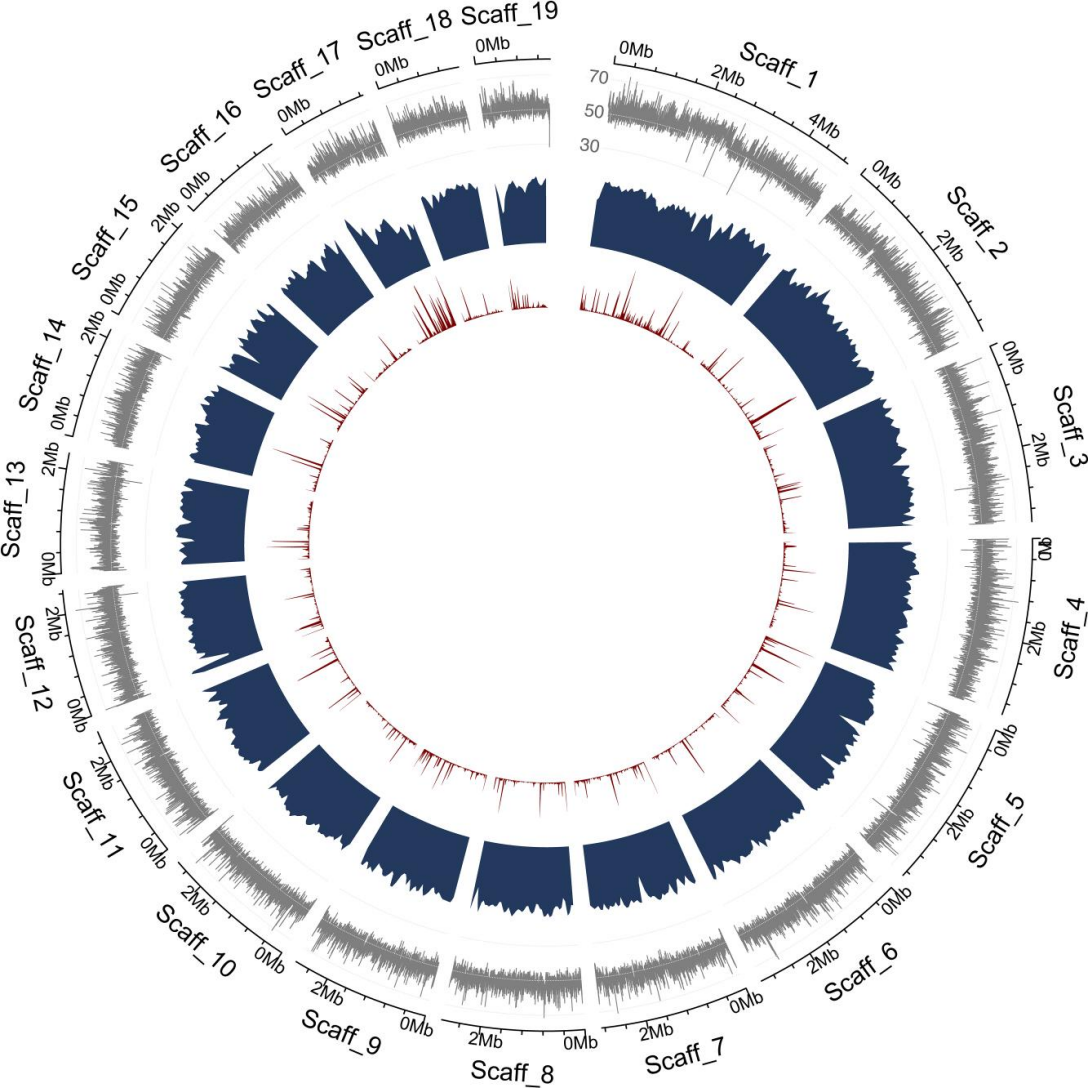
Circos plot summarizing the nuclear genome annotation of *C. elongata* SAG 216-3b. From outside to inside the tracks display per scaffold (Scaff): GC content (over 1-kb windows, outermost graphical circle in gray), gene density (second graphical circle in blue), and repetitive element density (innermost graphical circle in red).

Transcriptome-guided genome annotation yielded 14,811 gene models with an average length of 3.2 kb and one predicted transcript per gene model (Table 2), suggesting that the level of alternative splicing in the genome is very low. Of the 14,811 genes, 61% have annotated Pfam domains and 841 are predicted to carry a signal peptide for secretion. Moreover, 6.8% of the genome is composed of repetitive elements (Table 2), comparable to the 8.9 and 7.2% of repetitive sequences found in the genomes of *C. viridis* and *C. supellipsoidea* C-169 (Blanc *et al*. 2012; Kraege *et al*. 2024a). These 6.8% repetitive elements were annotated as TEs (3.1%), simple repeats (0.5%), small RNA (0.2%), or unclassified repeats (3.0%) (Table 2). Repetitive elements were evenly distributed across the genome with only a few repeat-rich regions (Fig. 5). A total of 1,494 (98.4%) complete gene models of 1,519 conserved BUSCO (Manni *et al*. 2021) in the chlorophyta_odb10 database were identified (Table 2), suggesting a highly complete genome annotation.

**Table 2.** Annotation features of the *C. elongata* SAG 216-3b genome. BUSCO assessment results are given as complete (C) and single-copy (S), complete and duplicated (D), fragmented (F), and missing (M). n is the number of complete gene models.

Genome annotation	
Repeat content (%)	6.8
Retrotransposons	2.9
DNA transposons	0.2
Small RNA	0.2
Simple repeats	0.5
Unclassified	3.0
No. gene models	11,923
Average gene length (bp)	3188
No. exons	124,306
Average no. exons per gene model	8
Average exon length (bp)	165
No. transcripts	14,811
Average no. transcripts/gene model	1
No. gene models <200 bp length	0
No. proteins with ≥1 Pfam domain	9004
No. proteins with signal peptide	841
**BUSCO (chlorophyta_odb10)**	**C: 98.4% [S: 80.4%, D: 18%], F: 0.4%, M: 1.2%, n: 1519**

## Supplementary Material

jkae294_Supplementary_Data

## Data Availability

Data for *C. elongata* Chodat & Jaag SAG 216-3b is available via the European Nucleotide Archive (ENA) under the study accession number PRJEB79308. Fastqc reports of raw data can be found in (Kraege *et al*. 2024b). The genome annotation outputs as well as the scripts used in this study are available on https://github.com/antonkraege/Celongata_Genome/tree/main. Supplemental material available at G3 online.
