# Supplementary material for: High quality genome assembly and annotation (v1) of the eukaryotic freshwater microalga Coccomyxa elongata SAG 216-3b
Source: G3 (Bethesda). 2024 Dec 13;15(2):jkae294. doi: 10.1093/g3journal/jkae294 (PMC11797067; doi:10.1093/g3journal/jkae294)
Supplement: jkae294_Supplementary_Data [file jkae294_supplementary_data.pdf]

Table S1. Summary of bioinformatics tools used for genome assembly and annotation.

Assembly		Annotation	
Tool	Version	Tool	Version
Raven	v1.8.1	RepeatModeler	v2.0.3
Juicer	v2.0	TransposonUltimate	v1.0
BWA	v0.7.17-r1188	TEclass	v2.1.3
3d-dna	v180922	RepeatMasker	v4.1.2-p1
Juicebox	v1.11.08	HiSat2	v2.2.1
Minimap2	v2.24-r1122	Braker	v2.1.6
Samtools	v1.10	Gffread	v0.12.7
Integrative Genome Viewer	v2.11.2	SignalP	v6.0
Tapestry	v1.0.0	BUSCO	v5.3.2
Blobtools	v1.1.1	R	v4.2.0
BLAST	2.13.0+	Circilize	v0.4.14
Kmer Analysis Toolkit	V2.4.2	InterProScan	v5.61
Mummer	C3.23		
